# Lectures on Diseases of the Eye

**Published:** 1839

**Authors:** 


LECTURES ON DISEASES OF THE EYE. By *John Morgan*, F.L.S. Surgeon to Guy's Hospital, and Lecturer on Surgery at that Institution. 8vo. pp. 221, 18 coloured plates. Highley, 1839.

THE modest announcement we shall quote, will explain the author's aspirations in publishing the present work.

"The following Lectures are published at the request of my pupils, and not from any wish, on my part, to appear before the profession, and the public, as an author of what will I fear be considered by them, as a very imperfect work, on the subject of Ophthalmic Surgery generally. This admission will, I trust, shield me from hypercriticism. Neither as a lecturer, nor as an author, do I put myself forwards as a competitor for professional fame and distinction, with those who have preceded me as the public instructors of their students on the science herein treated of, and as advocates for the connection of Ophthalmic with general Surgery; but having been repeatedly urged, not only in private, but publicly, by so many of my class to supply them by publishing my Lectures, with that, which they considered would be a short text-book for their guidance, after they entered into practice, as well as during their studies at the Guy's Hospital Eye Infirmary, I now do so in compliance with their wishes, in the hope of affording them a permanent and perfect reminiscence of those instructions, which I have spent so many proud and grateful hours in offering to them. I cannot refuse to take all the chances of good or evil which may await me, in thus acceding to the wishes of my young friends, although laying myself open, as I doubt not I am now doing, to the censure of many who, perhaps, from various causes may have expected something better than my present production.

My object has been to describe concisely and clearly, the more common and the more important diseases, to which the eye is subjected, with what experience has taught me to be the best general treatment, and to illustrate, as much as possible, the analogy between diseases of the eye and those of other parts of the body.

If a detailed account of *every* morbid appearance, connected with the component parts of the organ of vision, minutely and classically described, be required,

I must refer my readers to the works published on the subject by Travers, Lawrence, Mackenzie, and some others; I have no pretensions to oppose to the learned productions of those gentlemen the humble volume, which is now offered to the student of Ophthalmic Surgery."—*Pref.*

This confession would disarm criticism, and, even were it strictly true, a minute examination of defects would be ungenerous. But Mr. Morgan has not done justice to himself, and the work is one which others besides his students may consult. It is a plain, judicious, and practical treatise. It gives the pith of what is known and of what it is requisite to know, and avoids those minute details, which, however appropriate in systematic works, fail to sustain their claim to utility in the actual practice of ophthalmic art.

That the science is overlaid with terms, and that refinement has "gone the whole hog," cannot, we imagine, be denied. Simple effects of simple causes, analogous to what occurs at every turn in other regions of the body, have been baptised with jaw-disturbing names, and trivial appearances have formed the subject of chapters. Mr. Morgan admits this, though the passage we shall cite has been written with another object.

"The long established practitioner, in whose younger days this study was neglected, cannot perhaps be expected 'to go to school again;' his numerous professional avocations prevent his doing so. He takes up, however, in a leisure hour, a long spun treatise on Ophthalmic Surgery, (having no time either to attend Lectures on the Pathology of the Eye, or to avail himself of the practical knowledge to be obtained in the wards of an Eye Infirmary,) and in that voluminous production finds a surprising variety of terms descriptive of common diseases, which, if reference is had to the treatment of the same diseases occurring in the structures of other parts of the body, will be found to yield to the same class of remedies; but the multiplied names by which they are designated, bewilder him as a beginner so completely, that he gives up the subject in despair, under the soothing persuasion and consolation, that others far more distinguished than himself in his profession, are just as little, if not less, acquainted with eye diseases than himself." 4.

The farrago of terms bandied about by professors of ophthalmic surgery would be utterly absurd, were they not mischievous as well. But we quit this subject.

We shall skim lightly Mr. Morgan's book, and pick up here and there some practical remark from its surface. Connection in our quotations is out of the question. They merely embody a few scattered hints dropped by a man of plain good sense and information.

1. *General Rule in Treating Diseases of the Eye.*—"In active diseases of the Eye, there is one general rule, which you cannot remember too well,—it is 'never to rest contented with merely checking the progress of disease,'—never, if you think that you can prevent it, by pushing your remedies still farther,—never allow a disease of the eye to remain stationary. In other organs, where you have neither the transparency or integrity of a delicate tissue to preserve, nor an expanded sheet of nervous matter to protect from the slightest causes of disease, this may not be a point of any very material consequence. But, in active diseases of the eye, the delay of remedies for twenty-four hours will, in some cases, prove a sufficient cause for complete disorganisation of the organ.

Never, therefore, in any cases, or under any circumstances, rest satisfied with watching day after day the partial control of remedies in Ophthalmic disease.

Unless you are daily diminishing that disease, you are doing your patient more harm than good, by your treatment; for whilst you are watching and waiting for the more obvious effects of your half measures, the half-controlled disease will be insensibly extending to more important parts; and by the time you have determined upon altering the plan of treatment, either the functions of the retina will have been destroyed, or the condition of the transparent parts of the eye will have been permanently changed. Therefore, remember, that in allowing any active inflammatory disease of the eye to remain stationary for a day, you are endangering the safety of the organ. This rule may apply to all inflammatory diseases of important organs; but it is more particularly applicable to the organ of vision." 9.

2. *Distinction between Catarrhal Ophthalmia and Ophthalmia from Extraneous Bodies.*—Mr. Morgan judiciously observes, that these two affections are not unfrequently confounded, or one is mistaken for the other. His hints on their discrimination are simple and satisfactory.

In catarrhal ophthalmia we frequently find a distinct surface of inflamed conjunctiva upon that part of the membrane which covers the lids, and upon that part only. In those cases, where inflammation of the membrane arises from the irritation of a foreign substance pressing upon the part, this partial effect can never be produced; for the local irritant being placed between the conjunctiva of the globe and the conjunctiva of the lids, will press equally upon both; and, therefore, any appearance of increased vascular excitement observed in one part will be attended with a similar indication of disease in the other,—the foreign body pressing equally upon both surfaces will produce a degree of inflammation in the conjunctiva of the globe corresponding with that which is observed in the conjunctiva of the lids. In one case, then, both surfaces will be necessarily inflamed. In the other case it occasionally happens that one surface only will be the seat of disease.

In addition to the general inflammation of the conjunctiva of the globe following the introduction of an extraneous body, a copious secretion from the membrane and from the lachrymal gland is immediately and constantly poured out. In catarrhal ophthalmia this secretion is neither so profuse nor so long-continued as in the other case. Mr. Morgan admits that these diagnostic symptoms are not applicable to every case, but he contends that they are so to many.

3. *Treatment of Catarrhal Ophthalmia.*—One would imagine that if medical men could agree on any thing, it would be on the treatment of so common a disorder. The contrary is notoriously the case. We shall mention Mr. Morgan's plan of treatment, and leave our readers to judge for themselves of its propriety.

He commences by a brisk mercurial purgative, and, perhaps, an emetic. Then come general, (rarely), often local bleeding—a light shade, and avoidance of heat and light—*generally*, saturnine, or evaporating washes—*sometimes* warmth and moisture, *always* deference to the feelings of the patient—the introduction of some mild ointment between the lids at night—diaphoretics, and aperient neutral salts—in the chronic stage, blisters, to the temple or the nucha, and a collyrium of equal parts of aqua rosæ and vinum opii.

We would hint a single objection. So far as we have seen, warm applications have been more useful in the early stage, stimulants in the later, than Mr. Morgan represents them.

4. *Wrong and right Modes of examining an inflamed Eye.*—Mr. Morgan rather dwells on this matter. He presents three drawings, and matches them with three descriptive sketches. The thing seems trivial, is important, and we quote the passage which refers to it.

"The usual mode in which a bungler makes the attempt is represented in fig. 1. Each thumb is placed on the margin of the tarsal cartilages—pressure is made down upon the globe—the orbicularis contracts powerfully from sympathy—and thus the conjunctiva of the lids and of the globe are rubbed together as the lids are slid over the anterior part of the latter. The natural consequence of this mode of proceeding must be obvious; when two inflamed membranes are rubbed and pressed together, of course the diseased action will be increased; and, consequently, when a successful effort has enabled the operator to draw asunder the lids in the manner I have now described, it is found that the vascularity of the part is very greatly increased from irritation produced by previous pressure and friction.

This, then, is the most common mode of opening an inflamed eye improperly, and the consequences are always as I have stated.

The next diagram (fig. 2) represents a less common, though at the same time a far more injurious, mode of making the examination. It consists in the process of insinuating elongated finger-nails between the lid and the globe, and thus clawing the inflamed surfaces asunder. That such an operation must necessarily tend not only to produce a temporary excitement, but also a permanent increase of inflammatory action in the part will not be doubted. Yet I know from experience that this operation is practised too frequently to be passed over in silence on the present occasion. Look at these two diagrams, and see whether before you are twelve months' older, you do not observe an illustration of the error here represented.

Having now shown you the consequences of an unskilful attempt to open an inflamed and swollen eye, I will next describe to you the proper mode of performing this very simple operation.

Your object will be to *separate* the inflamed surfaces of the conjunctiva of the lids and globe at the time you are opening the eye, and to avoid making any pressure upon the part; this will be easily accomplished, unless excessive tumefaction of that part be an obstacle, by gently drawing the integuments of the lower lid downwards towards the cheek with the point of the fore-finger of one hand, and with the thumb or fore-finger of the other, drawing the skin covering the upper lid upwards towards the supra-orbitar ridge,—the diagram (fig. 3) represents what I have described. Be careful in opening an eye to avoid throwing a strong light on the part, as it sometimes renders the operation difficult, from the spasmodic contraction of the orbicularis palpebrarum, and in cases where the retina has been rendered morbidly irritable, a temporary increase of vascularity will generally be the consequence.

You may perhaps think that I have laid great stress upon a comparatively trifling subject, but experience will prove to you that minor points in your surgical practice are of more importance than you may now suppose." 26.

We quite agree with Mr. Morgan on the right method of examining the eye. But, in spite of his diagnostic suggestions, a difficulty may occur in determining whether there is not some little extraneous particle lodged beneath the superior palpebra. The mode of examination recommended will be insufficient to determine this, and eversion of the upper lid is requisite.

The apparatus of a probe, and of a good deal of pulling at the cilia, may often be entirely dispensed with, a quick jerk at the lashes being sufficient for the version of the tarsal cartilage. This should never be done until the simpler examination has been first made, and the actual amount of vascularity appreciated. However dextrously it may have been accomplished, it is certain to occasion suffusion of the conjunctiva.

5. *Pustular or Aphthous Inflammation of the Conjunctiva.*—This, as is well known, is considered by some, and, we think, on very insufficient grounds, the strumous ophthalmia. Be that as it may, we notice Mr. Morgan's account of the mode of vascularity of the pustules or aphthæ, for a reason which will be presently apparent. We should state, in limine, that Mr. Morgan compares the conjunctival pustules with the ordinary aphthæ of the gastro-intestinal mucous membrane. We now quote the passage to which we have referred.

"If seen at the very commencement, a minute whitish raised speck will be observed on the surface of the membrane, around which extremely minute bright red vessels radiate, in the form of a plexus, and are tortuous in their course, and occasionally in the *first instance* receive no visible supply of coloured blood from adjacent larger trunks. The reddened circle is perfectly distinct, and if occurring on the sclerotic conjunctiva, where its cellular connection is comparatively loose, it can be distinctly proved by moving the aphtha from side to side with the point of the finger, that no subjacent supply of red blood is sent to the part through its base from the vessels which lie behind it. After a time, conjunctival vessels, in the form of a fasiculus, are seen carrying red blood from the circumference of the globe to the aphtha, and then pour out their supply to its surrounding vascular zone; but not always in the first instance, for the zone is in some rare cases circumscribed and distinct. Now, of course, you know that aphthæ or pustules in other parts are surrounded by a red margin, and have been told that this arises from the passage of red blood from larger trunks into those minute ramifications of the vascular structures formerly carrying colourless blood. On examining the part, however, to see the causes of the change which has been going on, you see only a blush of inflammation, the vessels being too small to admit of examination without the aid of a strong magnifying power; but it is not so when inflammation attacks the conjunctiva, and reddens that membrane; for, under such circumstances, the fact is proved to you by ocular demonstration. But a still more interesting pathological fact appears also to be proved by one circumstance I have mentioned, viz. that in inflammation the change of the fluid contents of an artery from colourless to red blood does not necessarily depend upon the direct transmission of the red particles from a larger trunk into the dilated capillaries; that such change does take place is undoubted, but that the redness of an inflamed structure depends exclusively upon this mode of altering the colour of the component parts of the circulating medium is surely rendered more than problematical by the already described phenomena (to which others might be added, and) which present themselves so clearly to our notice during the commencment of the formation of a conjunctival aphtha. How can we suppose, in these cases, that the colour of the blood is changed in the vessels, unless we look for some cause in addition to that of the mere mechanical conveyance of the red globules from the larger to the smaller branches of an artery." 29.

We do not clearly see the drift of these observations. How the vascular zone is to get red blood, in any other way than through the medium of vessels which have brought it, is not obvious to our understandings. Mr.

Morgan does not appear to be aware that vessels which convey but a single row of coloured globules of the blood, will not be visible by the naked eye. Supposing such vessels to become dilated at their extremities by inflammation, the dilated portions will exhibit vascularity, while the undilated parts will be invisible. This appears to us the simple solution of the difficulty which perplexes Mr. Morgan. At all events it is more consistent with probability than the rather mysterious idea which he entertains, of some undefined alteration in the blood.

The treatment of this disorder may interest our practical readers more than discussions of the preceding description. The complaint is almost always troublesome, not unfrequently contumacious to the last degree. Any hints on its effective management will be gratefully received. First we subjoin one or two very sound remarks upon tonics.

"The different preparations of cascarilla, calumba, or bark, in combination with an alkali, more particularly ammonia, may be used with advantage, if judiciously given. In addition, an occasional purgative will be found highly beneficial; but strict attention to proper regulation of diet cannot be too strongly insisted on. In these, and indeed in all cases of disease, tonic medicines should be avoided when tonic diet can be substituted. If the digestive organs are performing their healthy functions, and you meet with no disturbance in the nervous and vascular systems, further than that which debility alone will occasion, regulate the diet of your patient, instead of loading his stomach, as too many are in the habit of doing, with bulky tonic drugs. Tonics and stimulants, in the way of medicine, may certainly be required sometimes; but not, I believe, as often as they are given, and they ought never to be employed in those cases where the different secretions from the body are vitiated or suppressed; but with a natural appetite and healthy secretions from the alimentary canal, tonic medicines will be found more beneficial to the compounder than to the patient." 32.

But, Mr. Morgan goes on to observe, the local treatment is generally the most important, and will consist, in severe cases, viz. when the disease is attended with acute surrounding inflammation of the conjunctiva, and when the aphthæ are numerous,—in these cases it will consist in lessening action by topical bleeding, and by setting up counter-irritation in the neighbourhood of the disease. Leeches or cupping may be applied on the temple. Blisters or issues afterwards may be necessary, only, however, in very severe cases, for generally an astringent collyrium will be sufficient to excite the vessels of the part to altered and healthy action. Various forms will answer equally well.

Either of the following formulæ will be found useful:—

℞.—Liq. plumb. subacet. ♏vj.

Aq. fontan. ℥xiv.

Liq. Opii sedativ. vel Vini Opii, ʒij.

M.

℞.—Argent. Nit. vel Zinci Sulph. vel Cupri Sulphat. gr. ij.
Aq. fontan. ℥x.
Vini Opii, ʒij.
M.

The Vinum Opii or Liquor Opii Sedativus undiluted, are also beneficial as collyria, more particularly in chronic cases.

The salts of lead are generally open to objection in cases of impending or actual ulceration. The lead is precipitated on the ulcerated surface, and may either injure by its adhesion, or, what is more probable, not accelerate reparation.

6. *Variolous Inflammation of the Conjunctiva.*—The observations on this head are so sensible, so much to the point, and so likely to be useful in every-day practice, that we will not allow them to slip by. Mr. Morgan describes Variolous Ophthalmia under two heads:—first, as it affects the conjunctiva of the lids only; and secondly, as to the consequences which follow the extension of the eruptive disease over the whole surface of the membrane.

*a.* In watching a case of small-pox, we observe, remarks Mr. Morgan, that during the eruptive stage the lids are swollen and closed, and in a short time matter exudes from behind them, and becomes encrusted on the palpebræ and eyelashes, agglutinating the lids and perfectly sealing the aperture between them. This swelling of the part arises from the formation of pustules on the subjacent conjunctiva, the surrounding tissues becoming inflamed as the eruption is becoming fully matured, and the purulent effusion, which occasions by its incrustation, adhesion, and closure of the palpebræ, is poured from the suppurating surfaces of the broken pustules beneath. When agglutination is complete, the matter no longer finding an outlet for escape, is, together with the lachrymal secretions, which now increase in quantity, confined between the lids and the globe, where, until proper measures are adopted to evacuate and insure a free and constant outlet for the morbid discharge, it must, of course, occasion and keep up continued irritation over its inclosing surface of inflamed mucous membrane. Additional suffering to the patient is necessarily the consequence of such a condition of the part. The symptoms complained of are sense of fulness, smarting pain, and burning heat, each increased by moving the globe in the orbit, or attempting to separate the lids. If these symptoms only, combined with the external indications which have been alluded to, are the most severe and prominent features of the complaint, and more particularly if it is found that the commencement and progress of variolous disease in the membrane hidden from view, is apparently keeping pace with that which is distinguishable in other mucous membranes, and on the surface of the skin—if we can see pure pus only discharging from the eyelids during the progress of the disease, and the swelling of the lids beginning to subside, with a diminished purulent secretion issuing between them, as a change concomitant with the alterations which take place in the pustules on the surface of the body, indicating the decline of the eruptive diseases,—then, even before the lids can be separated, we may generally anticipate a favourable result, for general and perfect acute Variolous Ophthalmia is distinguished by far more severe suffering.

As the complaint is subsiding, the swelling gradually diminishes, and in a short time the lids can be again separated, and the globe of the eye distinctly seen. It will then be found, that if those symptoms only have been present which have been mentioned, and if the disease in the eye has kept pace with that on the skin in its progress and convalescence under such circumstances, it will be found, that if the cornea has escaped, recovery will follow, without damage to the sight. A trace, however, of previous disease will be sometimes left, for the eyelids in the situation of the former pustules are sometimes disfigured by cicatrices, producing an uneven edge of the tarsus, which may remain for the rest of life. The cilia in many cases fall out, and are seldom restored. Trichiasis, or inversion of the eyelashes, is also a frequent consequence of variolous inflammation of the tarsal conjunctiva.

The treatment, though simple, is far from inefficient or unnecessary. The object will be to prevent the occurrence of any increase of irritation in the inflamed mucous membrane, from the confinement of increased lachrymal secretion and purulent effusion behind the agglutinated and closed eyelids. This will be accomplished by frequent tepid ablutions of water or milk and water. Mr. Morgan has generally found the following collyrium, injected warm between the lids, extremely useful and grateful to the patient:—

℞—Aq. Ros. ℥viiss.

Liq. plumb. subacet. ♏xx.

Liq. Opii. sedativ. ʒij.

M.

This, of course, can only be used after the incrustations have been removed, and a free passage established for the escape of the morbid secretions and injection of collyria. The removal of incrustations from time to time is a point of great importance. Considerable relief is often afforded by puncturing the pustules of the affected parts.

*b.* "Now, when instead of affecting the lids only, the pustular eruption extends over the sclerotic and corneal conjunctiva, the disease becomes dangerous in the extreme. We have, in addition to the formation of pustules, an acute inflammation of the whole membrane, and this general and pustular inflammation constitutes acute Variolous Ophthalmia.

You will recollect that the swollen state of the lids will quite prevent you from ascertaining whether the lids only, or the globe also have become affected. You will therefore ascertain the existence of acute Variolous Ophthalmia by the following symptoms:—Excessive lachrymal discharge, intolerance of light, even through the lids, pain on moving the globe in the orbit, continued pain, and sensation of sand or gravel between the lids. If you observe a bloody, greenish, offensive discharge, you may be sure the cornea is sloughing. These symptoms, then, indicating general and pustular inflammation of the external tunics, will lead you to anticipate the most serious possible consequences. The cause of danger in these cases must be obvious to you, when you consider that the disorder, as it affects the cornea, is precisely the same as when affecting the skin. In both cases, therefore, we have a small spot in which suppurative inflammation is set up, viz. a pustule. Ulceration follows, and sometimes sloughing; and wherever a pustule of small-pox forms on the transparent cornea, on that spot opacity to a greater or less extent will be produced by the cicatrisation of the consequent ulcer. If, therefore, a pustule be formed in the axis of vision, the functions of the organ will often be permanently disturbed or destroyed. If a small ulcer only be formed, and consequently a small spot of opacity be left, you will generally find that the sense of sight will be but little impaired. If a large superficial ulcer be formed by the pustules of confluent small-pox, and that ulcer leave behind it a cicatrix opposite to the pupil, the loss of sight is inevitable. If the disease be raging in its most virulent form, the sloughing process will form a large opening through the cornea, and allow the humours to escape. The globe in these cases will collapse, and, of course, the functions of the organ will be destroyed. If only a small ulcerated opening be produced, you will find that instead of a general evacuation of the humours, the aqueous only will escape, the iris will be pushed through the aperture, and thus prolapsus of the iris will be the consequence. If, again, the opening through the ulcerated cornea be too minute to admit the passage of the iris, it is found that the iris, in consequence of the escape of the aqueous humour, is pushed against the ulcerated aperture in the cornea, to which it adheres without protruding; thus producing an alteration in the relative situation of the membrane, which is called Synechia Anterior.

Sometimes the layers of the cornea, having been thinned by superficial ulceration, give way, bulge out, and thus form a permanently opaque projection, which is known by the term of Staphyloma." 40.

On the treatment, there is not, unfortunately, a great deal to be either said or done. In small-pox, extreme depletion can be seldom ventured on. But Mr. Morgan would seem to think such extreme depletion, à priori, called for by this species of ophthalmia. In the most acute forms of the complaint, if the constitution will bear it, no time should be lost in endeavouring to check the progress of the disease by general bleeding—local bleeding—mercurial purgatives—warm ablutions, to prevent agglutination,—placing the patient in a well-ventilated room, excluded from light. But if such active measures are forbidden, local bleeding and tepid ablution must constitute the full stretch of our tether. Mr. Morgan gives a parting caution:—Do not, he says, mistake the common effects of small-pox on the lids only for acute Variolous Ophthalmia. Do not bleed, either generally or locally, when the lids are only swollen and glued together, for this is the effect of pustular disease on the conjunctiva adjacent to the edges of the cilia; but when, in addition to this swelling and agglutination, you find continued pain—intolerance through the lids—profuse lachrymation—sensation of sand between the lids—and excruciating pain on moving the globe within the orbit,—then rest assured that by depletion only the organ can be saved, for such are the symptoms of suppurative and ulcerative inflammation of the cornea, and of acute inflammation of other tunics. Thus your constitutional treatment in Variolous Ophthalmia must depend, not upon the urgency of the local symptoms, but upon the effect which the disease in other parts has made upon the system generally.

*c.* Mr. Morgan goes on to paint the secondary pustular ophthalmia, which occurs after the lapse of two or three weeks, when the small-pox pustules on the skin have formed incrustations, which are falling or have fallen off. Danger may seem to have departed, the medical practitioner may be off his guard, when the conjunctiva of the globe is again invaded with the pustular eruption.

At this period, however, the disease in the eye will be of a much milder form, and will be characterised by the following symptoms. As the lids are not so much swollen, we can now see the cornea, the pustule on which will first show itself in the form of a small circumscribed opaque spot, surrounded by a hazy zone. We next find that the opaque spot increases in size, and forms in its centre a yellow speck. This central yellow speck or spot increases to form a perfect pustule.

If it should happen that several pustules form on the cornea at the same time, it will sometimes be difficult to distinguish the opaque spots separately; for their surrounding zones of hazy cornea will become mingled together to form a nebulous or clouded cornea. In these cases a minute examination will be necessary, to ascertain the exact nature of the disease. The other symptoms will be, as in the former case, redness of the conjunctiva of the sclerotic and lids—pain and intolerance of light. The disease, therefore, is the same in kind, but less in degree. Suppuration and ulceration are the most common consequences of the disease in this stage; the sloughing process rarely supervenes.

Mr. Morgan's plan of treatment is the same as before:—active and continued general depletion, which the patient can generally bear after the decline of the eruptive fever—local depletion—tepid washes—and a shaded, cool, and well-aired apartment.

It does appear to us that Mr. Morgan is rather timid in the use of stimulants. We cannot coincide with him in his almost unreserved employment of depletion. When ulceration actually exists, we do think that depletion is often injurious, rarely very beneficial. The nitrate of silver in solution, in ointment, or in substance, is then most commonly of signal service. Such, at least, is the result of our observation, and we cannot refrain from placing it in juxta-position with Mr. Morgan's, in order that our readers may compare and judge for themselves.

7. *Purulent Ophthalmia of Infants.*—Mr. Morgan dwells on the *best method of examining the eye of an infant.* He has already given us some hints on the way of going to work with an adult. The methods differ in the two cases.

"Now, the mode of separating the lids of a child, the subject of this disease, and the mode of performing the same operation in other diseases of the organ, and on the adult subject are totally different.

In many other diseases it is desirable that the inflammation of the conjunctiva should not be increased by any violence done to the part in an attempt to open the eye by artificial means, and therefore I pointed out the best mode of doing so in a former lecture. But in Purulent Ophthalmia of infants it will be quite impossible to effect your purpose in the way I then recommended. The conjunctiva is swollen and villous. You find chemosis of lids, and a cornea hid by protruding chemosis of membrane.

The best mode of exposing the globe will be by separating the lids quickly during the time the child is asleep; but it does not always happen that we are able to choose such a time for the operation, therefore another course of proceeding must be adopted. It consists in pressing the margins of the tarsi backwards over the globe, by placing the finger-nails against their anterior edges. No force is to be used—no pressure *downwards*—the finger-nails are not to be *hooked* under the lids, but placed against the anterior edges of the lids—which are to be separated by gentle pressure—the introduction of probes between the lids may sometimes be required to effect your object, or the use of a speculum oculi, if from the tumid state of the part, or the irritability of the child, or any other cause. Often, however, greater force is required than can be exerted by the finger only; we must rest satisfied in our diagnosis by the presence of other symptoms, viz. with the degree of tumefaction in the lids, and the character of the purulent discharge issuing from beneath them." 49.

We see nothing to claim attention in Mr. Morgan's treatment of this ophthalmia. But he offers a remark or two in reference to prognosis, which may not be altogether useless.

If, he says, you observe a bloody ichorous offensive discharge issuing from the eyelids, you may, without further inquiry, pronounce at once that the functions of the organ will be either seriously disturbed, or completely and permanently destroyed. That ichorous sanious discharge is produced in these cases by one cause, and by one cause only, viz. slough of cornea; and a sloughing of any large portion of the cornea in Purulent Ophthalmia of infants may be considered as synonymous with a total destruction of the organ. If, before you separate the lids, you perceive a deep yellow or a greenish yellow discharge, you will know that very acute inflammation is existing in the part. If the discharge be whitish, that white appearance will indicate a less degree of inflammation; and, if not preceded by a yellow effusion, you will generally find that the case will terminate favourably. But the only satisfactory evidence upon which you can found a correct prognosis will be afforded by an examination of the globe of the eye itself. When the cornea is clear and transparent, you may at once assure the parents that the organ is, in all probability, safe. The disease is then usually completely under your control. Whatever may be the condition of the sclerotic or palpebral conjunctiva, if the cornea is free from disease, the sight may be almost always restored. A hazy appearance of the cornea is not always an indication of an unfavourable result, for if the semi-transparent dulness of the membrane be only superficial—if merely confined to the conjunctiva of the cornea and its anterior layers—that haze will disappear under proper treatment. If the cornea should be perfectly opaque, ulceration or slough, to a greater or less extent, will follow, and consequently the eye will necessarily be damaged.

8. *Treatment of Strumous Ophthalmia.*—We may observe that strumous ophthalmia is that form which commences with extreme sensibility of the retina to light, constituting intolerance; redness and inflammation of the conjunctiva, with its consequences, follow. These phenomena may coincide with the ordinary marks of the scrofulous diathesis, or the latter may be imperfectly exhibited, nay absent. The complaint, as most surgeons have found to their cost, is frequently troublesome and obstinate to the last degree. We subjoin Mr. Morgan's plan of treatment.

Suppose a case in its early stage, when irritability of the retina is yet unaccompanied by continued redness of the conjunctiva. Our first object must be to clear the alimentary canal of its contents by purgatives. Some preparation of mercury, combined with a more active aperient, will be found the most useful medicine to begin with. Our next object will be to lessen irritability, which is dependent upon constitutional weakness, by a tonic plan of treatment; and at the same time to promote the healthy condition of the different secreting organs; for, in this complaint, as in Struma affecting other parts, you will meet with diminished power in the system, increased nervous irritability, and vitiated or suppressed secretions. When continued inflammation of the conjunctiva *does* exist, leeches may be necessary. But they should be sparingly used, and blisters, if possible, avoided. A warm bath is often serviceable, and in very obstinate cases the exhibition of small doses of opium, antimony, and mercury in combination given every night may be required: these, however, are usually cases where we find a hazy cornea, and minute red vessels overshooting its margin, or covering its whole surface; here, the safety of the organ is threatened, unless the system is made to feel the influence of mercurial action, and, now and then, the establishment of continued nausea must be had recourse to, to lessen the surrounding inflammatory action.

We have found more benefit from leeches, than Mr. Morgan would seem to have observed. A single leech applied to the temples, every or every other night, has often seemed to us extremely beneficial. In this, as in other instances, Mr. Morgan appears timid in the use of stimulating applications.

9. *Pterygium Pingue.*—Occasionally, writes Mr. Morgan, a spurious form of Pterygium occurs in old people. This consists in the formation of small yellow steatomatous granules, which are situated beneath the conjunctiva. The disease has been called Pterygium Pingue. It is totally distinct from the other complaint, and occurs almost always in elderly persons, never requiring removal or medical treatment. There is one distinguishing mark, which separates this from true pterygium. The latter is always of a triangular or pyramidal form; Pterygium pingue is circular or irregular.

10. *Conjunctiva of the Cornea proved to exist by Chemosis.*

"In some cases of Chemosis an appearance presents itself, which must be equally interesting to the anatomist and the pathologist; as it affords a perfect proof to the former, of the separate existence of a structure, which perhaps he might not be able by dissection to detect, and to the latter a guide for the treatment of his patient. I mean the appearance of Chemosis of the Corneal Conjunctiva, by which any attempts at argument intended to prove, from the apparent impossibility of separating the parts in the healthy subject, that the conjunctiva does not form an anterior covering to the cornea, are at once set at rest." 102.

11. *Precautions to be observed in applying Nitrate of Silver to the Eyelids.*—In making use, says our author, of the argenti nitras, or any other astringent, whether fluid, solid, or unctuous, to a diseased eyelid; first evert that lid, holding it well away from the globe; dry it by passing a piece of linen over its conjunctival surface, and be ready the moment you have dried it to apply your astringent, after having done which, immediately dry it again, and apply some mild unirritating ointment, wipe this off, and apply it once more, and you will then be almost certain that whatever may have been applied to the conjunctiva of the lid will not reach that of the globe, the object of preventing which must be obvious to you, when you consider the effect your strong local applications would otherwise produce upon a continuous surface of a healthy membrane.

12. *Distinctions between Natural Colouration of the Iris and Iritis.*—Every body knows that, under the influence of inflammation, the iris changes in colour. But, as Mr. Morgan succinctly puts it, it occasionally happens that in the same individual the iris is naturally of a different colour in each eye; that in one the iris is blue; in the other green. Sometimes an iris will be half green and half blue; in which case no mistake can be made, as this appearance is never produced by inflammation; but in those who possess two irides of different colours, it may occasionally happen that common acute inflammation of the conjunctiva might be mistaken for a case of pure iritis; in both you will have intolerance of light, pain, and an altered colour of the iris. Conjunctival inflammation is frequently combined with iritis, and the sclerotic zone of pink vessels will therefore in these cases be obscured or concealed beneath the tortuous vessels of the conjunctiva; so that an injected and vascular conjunctiva with a discoloured iris will be the diagnostic marks of the nature of the complaint. If it should ever become a matter of doubt whether under such circumstances the discoloured iris be the consequence of inflammation or of other causes, you will at once be able to determine by the condition of the pupil and the comparative brilliancy of the tunic; the colour may deceive you, the appearance of increased vascularity may lead you into error, but loss of brilliancy and a contracted or an irregular pupil which dilates and contracts slowly, are marks of distinction which are characteristic of iritis and of iritis only. By these symptoms, then, you will always be able to determine whether the iris is the seat of disease in those patients in whom the difference of colour might lead you into error.

13. *Diagnostic Marks of Syphilitic Iritis.*—We are quite sure that Iritis is seldom a *pure* secondary symptom. It usually occurs where mercury has been employed, sometimes where it has been grossly abused. The writers on ophthalmic surgery, lay down certain criteria of syphilitic iritis. The following are the characters insisted on by Mr. Morgan:—

Syphilitic Iritis presents precisely the same appearance under all circumstances; whether it accompanies the papular eruption or the elevated scab of rupia prominens, or whether it be found in combination with the squamous eruption, or uncombined with any cutaneous affection whatever; under all these circumstances the appearances will be as follows:—

The first symptom, as in common iritis, will be pain and intolerance of light; generally exacerbations of pain at night, and comparative ease during the day, and the pupil contracted and thickened, and generally drawn upwards and towards the inner canthus. The conjunctival vessels are generally unaffected, but the zone of sclerotic vessels around the cornea is always formed from the commencement of the disease. The colour of this zone in Syphilitic Iritis, is highly characteristic of the nature of the disease; it is not pink, but cinnamon or rusty brown-red; this is a good diagnostic mark of distinction between common iritis and Syphilitic Iritis.

In a short time patches of lymph are deposited on the anterior surface of the iris, and adhesions are formed at a very early period between the pupil and the capsule of the lens. The adhesive matter which is poured out, is sometimes, in these cases, of a reddish hue, and the effusion is more rapid and abundant than in common iritis, consequently the adhesions of the pupil are more extensive. Mr. Morgan adds, that the nocturnal aggravation of pain in the orbit, and in the globe, is more frequent in the syphilitic than in any other form of iritis. But, after all, the great diagnostic mark is the existence, antecedently or concurrently, of other venereal symptoms.

14. *Treatment of Arthritic Iritis.*—We subjoin Mr. Morgan's directions, without pledging ourselves to entire concurrence with him.

In pure arthritic iritis, mercury is seldom necessary, sometimes hurtful.

“The remedies for Arthritic Iritis are perfectly inert in both common and syphilitic inflammation of the tunic. Those remedies are as follows:—General and active antiphlogistic measures are hardly ever required. If, however, there be pain extremely severe, and febrile excitement, it may be necessary to abstract blood from the arm; but generally, local bleeding by cupping on the temple and the free application of leeches will prove sufficient. Counter-irritation, effected by the application of blisters, will also be found highly useful.

If intolerance of light is severe, the exclusion of it, by a green shade or veil, will of course be necessary; tepid fomentations are more useful than the application of cold. The medicines which you exhibit in the first instance will be given with a view of acting freely upon the bowels, and thus clearing the alimentary canal of its contents.

You afterwards administer those remedies which are found to be beneficial in cases of acute or chronic rheumatism in other parts. You are aware, I suppose, that in some cases of rheumatism the exhibition of bark has been found useful. This medicine is an exceedingly good one in rheumatic iritis, particularly when combined with an alkali. The following will be found a useful formula:—

℞.—Pulv. Cinchon. gr. v.

Sodæ Carb. ℈j.

Fiat pulv. ter in die sumend.

In all cases, restore or insure healthy and natural secretions by antimonials and small doses of mercury. Use mercury in these cases, not to act as a poison by affecting the salivary system, but as an alterative to increase and improve the system generally. Whilst you are giving these medicines, the patient is to avoid all stimulants in diet, such as wine, malt liquor, and animal food.

Now it will occasionally happen, that a chronic form of inflammation will remain after the more urgent symptoms have subsided, and in some few instances it may be necessary to affect the system with mercury; but these are not pure cases of Arthritic Iritis,—they are cases in which common chronic inflammation supervenes upon the other disease. The appearance of the eye partakes more of the character of common iritis; the iris remains dull, the zone approaches the cornea, and there is pain in the globe. In such mixed cases mercury may be necessary.” 150.

15. *Principles which should regulate the Administration of Mercury.*—We quote the following observations because we think them exceedingly just, and because we also think that the error they deprecate is one too frequently committed.

“When we give mercury in ophthalmic, as well as in other diseases, with a view of what is called affecting the system, we are too much in the habit of estimating the extent of its operation by the presence of mercurial fætor in the breath, inflamed and spongy gums, or profuse salivary secretion. It is too much the custom to push on the remedy until one or other of these three signs of its action shall be manifested.

It is true that they are proofs of its specific operation upon the body; but it is equally true that the system may be affected without any of these symptoms; and that consequently a powerful operation upon disease may be produced without salivation.

In many venereal affections which would yield to the sparing and judicious use of mercury, ptyalism will increase the disorder, so it is in inflammation of the sclerotic. We sometimes find that the disease is appearing in its most severe form when the system is saturated with mercury, and disappearing under your remedies as ptyalism is subsiding; yet in many cases the future exhibition of the remedy, in small doses, will be absolutely necessary for the permanent cure of the disease.

Whilst you are giving these smaller doses, recollect that you are to be guided by the two following rules:—

If the disease is yielding, although no red line is to be seen on the gums, you are not to increase your dose of mercury, with the view of affecting the salivary system. The effect of the remedy upon the disease is a sufficient proof that the system is sufficiently affected.

If, on the other hand, the disease is stationary, although the gums are affected, and salivation is commencing, you are not to push your remedy still farther, in the hope of being able to control the morbid condition of the sclerotic, by drenching your patient with mercurials. The slightest indication of a mercurial action upon the salivary organs, will assure you that you have gone far enough. If you excite ptyalism, you will increase the original disease, and lay the foundation for others of the most serious nature. When the disorder is manifestly under the influence of your treatment, never think of trying to produce inflamed gums, but give mercury as sparingly as possible." 156.

All this seems to us to be so judicious, so conformable with reason, and so consistent with fact, that we entreat the attention of our readers to it. Nothing can be more erroneous than the idea that salivation is requisite as a test of mercurial action. If the disease is yielding, what would any one have more? If it is not yielding, salivation may be beneficial or injurious, according to the nature of the circumstances and the case. In one instance, the amount of constitutional affection implied by salivation may be highly serviceable—in another, absolutely the reverse.

The rule we conceive to be—to look on salivation as an evil in fact, and an exception in practice. It should not be sought as a matter of course, but only under the pressure of particular indications.

It may be asked, what criterion we possess of the sufficiency of dose or of aggregate quantity, in the exhibition of mercury, if salivation is not to be solicited. The answer is obvious. On the one hand the diminution of the disorder or disease—on the other, the employment of that amount of the medicine which experience tells us to be usually enough—these are the safest, and really most practical standards.

16. *Treatment of Obstructed Nasal Duct.*

"There are two modes of dilating a strictured nasal duct without making a wound in the face; the first recommended, and I believe, first practised by Mr. Travers, consists in passing a punctum probe through the lachrymal duct into the sac, carrying it, after it has fairly entered that part, almost directly downwards into the nose, and thus clearing a small channel for the passage of the fluid which collects above. Mr. Travers recommends injections in these cases, made through the puncta by means of the introduction of Anel's finely-pointed syringe tubes; my practice consists in passing a curved probe *upwards* through the duct, an operation which, although it may require a little practice to perform it with facility, is, so far as my experience has gone, most satisfactory in its result. No force must be used in introducing the instrument, the point of which having been carefully fixed in the lower opening of the duct, after being carried under the inferior turbinated bone, is to be gently pushed upwards into the sac, through the stricture, by depressing the handle of the probe or sound, keeping the convex part next to the handle upwards; then, after having established a free passage through the canal from below, I generally inject tepid water from day to day, by means of a small catheter, introduced in the same manner as the sound, and attached to an Anel's syringe. By following this plan, suppuration may sometimes be prevented, but at all events a fistulous opening in the face is prevented, if the disease is confined to the membrane, (although an abscess may have formed in the sac,) in consequence of the prevention of any accumulation there, from frequent injections." 220.

Mr. Morgan *never* passes a style.

Most surgeons, we believe, will agree that the passing of a probe from the punctum (necessarily a very small one) is, in the great majority of cases, useless—that the passing of a probe from the nose is often serviceable, nay effectual—but that the total abandonment of the style is injudicious.

17. *Treatment of Entropeon.*—When of long standing, the disease requires a surgical operation. This, in cases where the inversion is not very great, consists in the division of the tarsal cartilage, at the outer canthus, keeping the divided parts afterwards separate during the healing process; but, in the severest chronic forms of the complaint, to insure a permanent and radical cure, either the whole of the cartilage must be excised, or a flap made by a double vertical section through the part, which is to be kept everted long enough to prevent union by adhesion, by means of threads passed through the edges of the flap, and attached by means of strips of adhesive plaister to the adjacent integuments. This operation was introduced by Mr. Guthrie.

"I have succeeded," adds our author, "in some cases remarkably well with Mr. Guthrie's operation, but in others have failed, and been obliged to excise the whole cartilage afterwards, which I now generally do, in the most aggravated forms of the disease, when of long continuance.

The removal of a portion of integument from the lid, and bringing afterwards the divided edges of the wound together, so as to draw the cartilage into its natural situation, by thus tightening the skin of the lid, has been recommended; but I have generally found the advantage which is derived from this plan of treatment only temporary, and for the most part the same may be said of the application of caustic to the integument covering the lid, made with a view of forming a cicatrix, and consequently of producing contraction of the surrounding soft parts." 214.

We have cured two very obstinate cases by the removal of a large quantity of skin from the palpebræ, but we have, also, like Mr. Morgan, seen this operation fail.

18. *Treatment of Ectropeon.*—The eversion is usually produced, writes Mr. Morgan, by cicatrices in surrounding parts, and at first it would appear, that the removal of the cicatrised integument, which immediately afterwards allows the lid to be brought up in most cases, would easily effect a permanent cure; but it is found that subsequent contraction takes place in almost every case, and something more, therefore, is required; the removal, however, of the cicatrix and adhesions to deep-seated parts, must always be the first step in the operation for a perfect and permanent cure, which may be effected in two ways, first, by cutting out a central triangular portion of cartilage, the base of the triangle being at the edge of the lid, bringing the parts together afterwards by suture, and preventing the union of the wound, occasioned by the removal of the cicatrix by adhesion, so as to allow the adhesive process in the cut edges of the cartilage to go on undisturbed by a subsequent tendency to contraction in the parts closed over, and covering the situation of the former cicatrix. This operation will be applicable only where the eversion is slight; but in the worst cases where eversion is excessive, in addition to the removal of the cicatrix, another means of remedy must be had recourse to, which may either be combined with the excision of a triangular portion of cartilage or not, according to the condition of the part; it will, however, generally be necessary when the cartilage is permanently altered in its shape by long eversion. The operation necessary to accomplish a perfect cure consists in patching a portion of continuous integument upon the gaping surface of cellular membrane, exposed by the removal of the cicatrix.

He adds, as the result of his own experience:—

"By this Taliacotian operation, I have not, to my recollection, failed in curing the deformity in any remediable case. The detached portion which you transpose is to be confined in its new situation by small metallic (platinum) sutures, and a light bread-and-water poultice used as a dressing. It rarely happens that more than one small strip of adhesive plaister under the poultice is required to assist in keeping the part in its place, if the sutures are properly applied; and sometimes a poultice will be all the dressing necessary." 216.

It must obviously be a severe case, which requires so severe a remedy.

We have picked out a large number of practical hints from this volume. Its pretensions are not great, but it is well calculated to effect the objects of its author. The latter bears a high character with his pupils, and these lectures will probably be favourably received by the profession.

